# Au Nanoparticles as Template for Defect Formation in Memristive SrTiO_3_ Thin Films

**DOI:** 10.3390/nano8110869

**Published:** 2018-10-23

**Authors:** Nicolas Raab, Dirk Oliver Schmidt, Hongchu Du, Maximilian Kruth, Ulrich Simon, Regina Dittmann

**Affiliations:** 1 Peter Grünberg Institut 7 and JARA-FIT, Forschungszentrum Jülich, 52425 Jülich, Germany; n.raab@gmx.net; 2 Institute of Inorganic Chemistry, RWTH Aachen University, 52074 Aachen, Germany; oliver.schmidt@ac.rwth-aachen.de (D.O.S.); ulrich.simon@ac.rwth-aachen.de (U.S.); 3 Ernst Ruska-Center for Microscopy and Spectroscopy with Electrons, Forschungszentrum Jülich GmbH, 52425 Jülich, Germany; h.du@fz-juelich.de (H.D.); m.kruth@fz-juelich.de (M.K.)

**Keywords:** resistive switching, memristive devices, memristor, SrTiO_3_, Au nanoparticles

## Abstract

We investigated the possibility of tuning the local switching properties of memristive crystalline SrTiO${}_{3}$ thin films by inserting nanoscale defect nucleation centers. For that purpose, we employed chemically-synthesized Au nanoparticles deposited on 0.5 wt%-Nb-doped SrTiO${}_{3}$ single crystal substrates as a defect formation template for the subsequent growth of SrTiO${}_{3}$. We studied in detail the resulting microstructure and the local conducting and switching properties of the SrTiO${}_{3}$ thin films. We revealed that the Au nanoparticles floated to the SrTiO${}_{3}$ surface during growth, leaving behind a distorted thin film region in their vicinity. By employing conductive-tip atomic force microscopy, these distorted SrTiO${}_{3}$ regions are identified as sites of preferential resistive switching. These findings can be attributed to the enhanced oxygen exchange reaction at the surface in these defective regions.

## 1. Introduction

Resistive switching oxides are highly promising candidates for active materials in future non-volatile memories [1,2] and neuromorphic circuits [3]. Although device materials typically require unparalleled levels of purity and perfection, the presence of donor-type point defects, such as oxygen vacancies, has been identified to be crucial for the operation of redox-based memristive devices [1,4,5]. Associated with this, oxygen-deficient thin films exhibit significantly reduced forming voltages [6,7,8,9]. For the resistive switching model system SrTiO${}_{3}$, it has been furthermore shown that even the cation stoichiometry strongly influences the device performance [10,11]. Moreover, numerous experimental hints revealed that extended defects such as dislocations [12,13], grain boundaries [14] and stacking faults [15] promote the switching process because they might act as nucleation points for filament formation. Therefore, methods to modify the defect density locally could be employed to engineer the filament position in memristive devices. Methods to influence or modify the filament position are highly attractive, since the stochastic filament formation process is a considerable source of variability in memristive devices. So far, the filament position has been engineered by electrode shaping [16] or by the adjustment of the device etching procedure [17]. In this manuscript, we present an approach to provide preferential switching and forming sites in memristive SrTiO${}_{3}$ thin films by employing Au nanoparticles (AuNPs) as defect nucleation centers. It has been shown in the past that Y${}_{2}$O${}_{3}$ and AuNPs, which were introduced into YBa${}_{2}$Cu${}_{3}$O${}_{7-x}$ thin films during thin film growth, resulted in columnar defects that acted as flux pinning centers and increased the critical current densities in magnetic fields [18,19]. Furthermore, AuNPs have been employed as defect formation centers to engineer the optical properties of SrTiO${}_{3}$ thin films [20]. In their work, a continuous Au layer was transformed into AuNPs by annealing-induced dewetting [21]. In this work, we employ chemically-synthesized AuNPs [22] as defect formation centers in SrTiO${}_{3}$ thin films. This chemical approach offers the possibilities to control the nanoparticle density and to fabricate ordered arrays of AuNPs on the substrate in various ways [23,24]. We studied in detail the epitaxial growth of SrTiO${}_{3}$ on AuNPs with various densities and investigated the impact on the growth mode, the defect density and the local conducting and switching properties.

## 2. Results

### 2.1. SrTiO${}_{3}$ Growth on Au Nanoparticles

**Figure 1:**
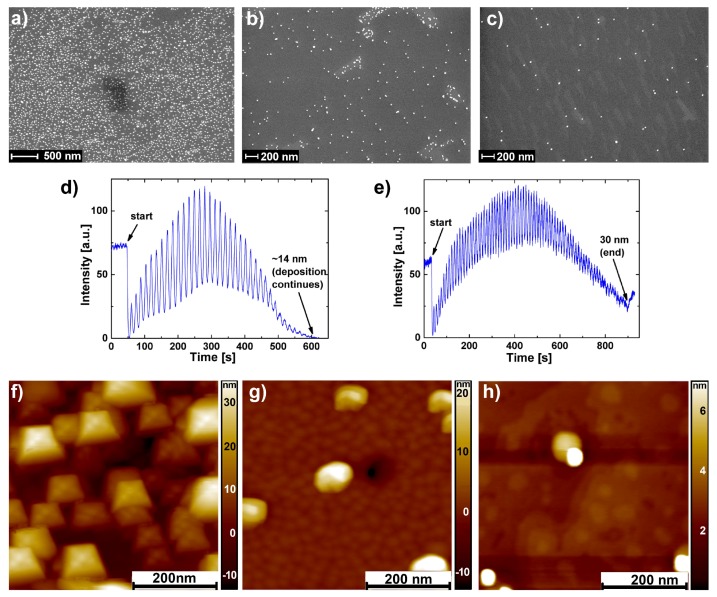
Growth of 30-nm stoichiometric SrTiO${}_{3}$ on Nb:STO substrates covered with various densities of AuNPs. (**a**–**c**) SEM images of the Nb:STO substrates with decreasing density from high (a) to low (c). (**d**) Evolution of the reflection high energy electron diffraction (RHEED) specular spot intensity for medium AuNP density. (**e**) Evolution of the RHEED specular spot intensity for low AuNP density. (**f**–**h**) Corresponding topography images of the resulting 30-nm stoichiometric SrTiO${}_{3}$ thin films with decreasing AuNP density from high (f) to low (h).

0.5 wt%-Nb-doped SrTiO${}_{3}$ (Nb:STO) single crystal substrates were covered with different densities of AuNPs, adjusted by the concentration of AuNPs in the solution. Details about the fabrication procedure of AuNP can be found in the “Materials and Methods” section. The corresponding scanning electron microscopy (SEM) images of the Nb:STO surfaces are depicted in Figure 1 for high density (a), medium density (b) and low density (c) AuNPs.

Thirty nanometer-thick SrTiO${}_{3}$ thin films were grown on AuNP-covered Nb:STO substrates by pulsed laser deposition (PLD), which was monitored by reflection high energy electron diffraction (RHEED). No RHEED intensity oscillations were observed for the sample with high AuNP density. However, for samples with medium and low AuNP densities, RHEED oscillations were observed throughout the deposition of the first 14 nm (Figure 1d) and the entire 30-nm thickness (Figure 1e), respectively. The RHEED oscillations therefore indicated a change of the growth mode from layer-by-layer to island growth at 14 nm for the samples with medium AuNP density. The corresponding topography images of the resulting thin film surfaces are depicted in Figure 1f–h. With high AuNP density, the surface exhibited a 3D topography of rectangular structures with a similar size and different heights (Figure 1f). It has to be noted that the observed structures were not caused by an imaging error of the utilized cantilever, as measurements with different cantilevers resulted in a similar topography. With medium AuNP density (Figure 1g), the overall topography exhibited small islands with frequently large, approximately rectangular hillocks with heights between 15 nm and 20 nm on top of these small islands. With low AuNP density (Figure 1f), a topography of a smooth step-terrace structure with spherical hillocks of 15 nm–20 nm height was observed. An additional elevated region with a height of approximately 9 nm can be observed next to the spherical hillocks in the center of the topography image.

With high AuNP densities, the growth mode was solely 3D growth, indicated by the absence of RHEED intensity oscillations and the rough topography depicted in Figure 1f. With medium AuNP density, the growth mode showed a transition from layer-by-layer to 3D growth mode, indicated by the vanishing RHEED intensity oscillation after the deposition of 14-nm SrTiO${}_{3}$ (see Figure 1d). With low AuNP density, the growth mode showed layer-by-layer growth throughout the entire deposition (see Figure 1e). However, a transition to multilayer growth took place since the second layer nucleation is visible on the step-terrace structure in Figure 1h.

It is important to note that the employed parameters for thin film growth resulted in stoichiometric SrTiO${}_{3}$ thin films with high crystalline quality [25] on bare Nb:STO substrates. Therefore, the change of the growth mode and the defective regions in the SrTiO${}_{3}$ thin films on AuNP-covered substrates have to be attributed to the influence of the AuNPs on the SrTiO${}_{3}$ growth. Although a direct correlation between the AuNPs on the substrate and the hillocks on the SrTiO${}_{3}$ thin film is not possible, it can be stated that the roughness of the SrTiO${}_{3}$ thin films increased significantly with the AuNP density.

**Figure 2:**
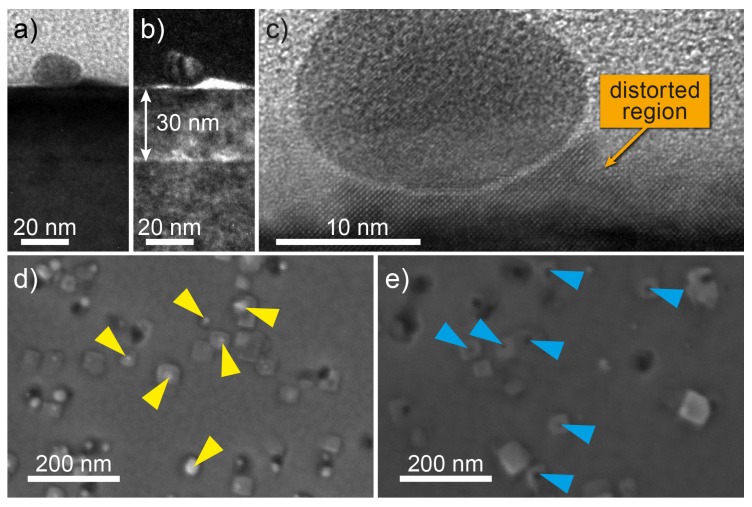
(**a**) Bright-field and (**b**) dark-field TEM image of a low density AuNP SrTiO${}_{3}$ sample in the vicinity of a AuNP. (**c**) TEM image of a AuNP with a distorted SrTiO${}_{3}$ region next to and underneath it. (**d**) SEM image of a sample with medium AuNP density after SrTiO${}_{3}$ deposition. The bright spots indicated by yellow arrows are assigned to AuNPs. (**e**) SEM image of a sample with medium AuNP density after etching with iodine-potassium iodide solution. The dark spots marked by blue arrows indicate holes in the SrTiO${}_{3}$ thin films.

To elucidate the composition and the origin of the hillocks shown in Figure 1, transmission electron microscopy (TEM) measurements were performed on the SrTiO${}_{3}$ thin films with low AuNP density. Figure 2 shows a bright-field (a) and a dark-field (b) TEM image. The interface between the Nb:STO substrate and the 30-nm SrTiO${}_{3}$ thin film, as well as the interface between the SrTiO${}_{3}$ thin film and the amorphous carbon protection layer, deposited by sputtering prior to the preparation of the TEM lamella, are clearly visible. An AuNP, with an approximately spherical shape and a diameter of around 15 nm, was located on top of the 30-nm SrTiO${}_{3}$ thin film. High resolution TEM (HRTEM) depicted in Figure 2c revealed an undisturbed SrTiO${}_{3}$ crystal structure and thus no extended defects beneath the AuNP. However, areas with distorted SrTiO${}_{3}$ existed next to and underneath the AuNP.

In order to investigate whether the AuNPs were located also at the surface for samples with medium AuNP density, we etched the surface with iodine-potassium iodide solution, which preferentially etches Au and thus AuNPs. The SEM images of the SrTiO${}_{3}$ thin film prior to and after the etching process are depicted in Figure 2d,e, respectively. The surface prior to the etching process was dominated by rectangular structures, which may correspond to the distorted SrTiO${}_{3}$ area Figure 2c, with bright dots on these structures. The rectangular structures were preserved, whereas the bright spots vanished, leaving dark spots behind. This confirms that the AuNPs were located on top of the distorted SrTiO${}_{3}$ islands for the sample with medium AuNP density, as well, and that the AuNPs were not overgrown by the SrTiO${}_{3}$ thin film, but floated to the surface during the SrTiO${}_{3}$ deposition.

Considering that the melting temperature of Au is in the vicinity of the deposition temperature (800 ${}^{\circ }$C), the mobility of the Au atoms is sufficiently high to enable a diffusion to the surface during deposition in order to reduce the surface energy. This results in the observed AuNPs on top of the SrTiO${}_{3}$ thin film. There were no extended defects in the SrTiO${}_{3}$ in the vicinity of AuNPs; however, distorted SrTiO${}_{3}$ areas were observed underneath and next to the AuNPs. A possible explanation for the occurrence of distorted regions might be that the sticking coefficients for TiO${}_{2}$ and SrO might be much lower on Au than on SrTiO${}_{3}$ and might strongly differ from each other. As a result, cation-deficient, strained SrTiO${}_{3}$ was formed in the vicinity of the AuNPs. These cation vacancies might be charge compensated by oxygen vacancies and result in a region deficient in both cations, as well as anions. Since oxygen vacancies have no significant influence on the lattice constant in STO [26], it is less reasonable to assign the observed distortion solely to a local oxygen deficiency. It is therefore suggested that the observed distortion has to be attributed to both cation and oxygen vacancies, similar to the structure observed by transmission electron microscopy within filaments induced in SrTiO${}_{3}$ devices by electroforming [27].

### 2.2. Nanoscale Switching Properties

In order to investigate how the local microstructure influences the resistive switching properties of the SrTiO${}_{3}$ thin films, we performed local conductive atomic force microscopy (LC-AFM) measurements. This method provides us with maps of the current between the thin film surface and the conducting Nb:STO.

**Figure 3:**
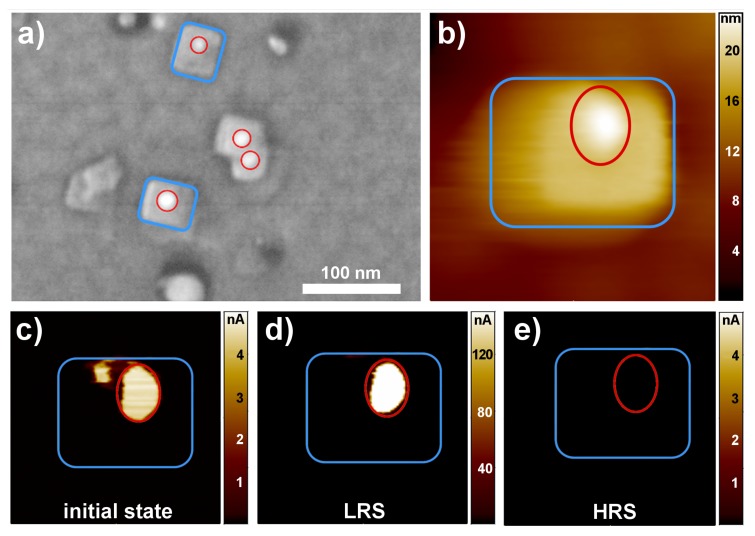
Type I resistive switching directly at the AuNP. (**a**) SEM image with characteristic rectangular structures (blue) and AuNPs (red) as the bright spot. (**b**) Topography image of a 30-nm stoichiometric SrTiO${}_{3}$ thin film, containing both a rectangular structure and an AuNP. (**c**–**e**) Current images measured with a read out voltage of +0.5 V in the same area as (b). For the initial (c), the low resistive state (LRS) after (a) a scan with +2 V (d) and the high resistive state (HRS) after (a) scan with −2 V (e). All local conductive atomic force microscopy (LC-AFM) images were taken with a scan size of 100 nm × 100 nm.

For that purpose, the surface was first scanned with a read-out tip voltage of +0.5 V to record the local conductivity distribution of the initial thin film surface. Afterwards, the film surface was scanned with +2 V, and a read-out scan was performed to record the impact of the positive biasing on the local conductivity distribution. Subsequently, the film surface was scanned with −2 V, and a read-out scan was performed to record the impact of the negative biasing on the local current distribution. In the initial state, a strongly-enhanced conductivity was observed in certain regions (Figure 3c). With positive biasing of the thin film surface, the conductivity in these regions increased further (Figure 3d), whereas it decreased by negative biasing (Figure 3e). This is in agreement with the so-called “eightwise switching” we observed in our Nb:STO-SrTiO${}_{3}$ -Pt devices [28]. For this switching polarity, it is proven that anodic oxidation induces the release of oxygen gas and an increase of oxygen vacancies within filament regions in the SrTiO${}_{3}$ thin film, resulting in an increase of the conductivity of the devices [29,30]. In turn, negative biasing leads to a reoxidation of the filament and a decrease of the conductivity. For our Nb:STO-SrTiO${}_{3}$ -Pt devices, we revealed that the oxygen after switching to the low resistive state (LRS) was partly stored within the grainboundaries of the Pt top electrode. During switching to the high resisitive state (HRS), the oxygen is partly reincorporated from the Pt top electrode and partly supplied by oxygen or water vapor present in the environment [30]. It has been reported in the literature that the water meniscus present at the AFM tip even in vacuum conditions can supply oxygen for thin film oxidation during negative biasing [31,32,33]. We therefore assign the source of oxygen for the LC-AFM oxidation process in our thin films (Figure 3e) to the water meniscus present at the AFM tip.

While the SrTiO${}_{3}$ surface of our conventional Nb:STO-SrTiO${}_{3}$ -Pt devices was homogeneously insulating after the removal of the Pt electrode in the initial state and conducting filaments arose only after forming with positive bias [28], our SrTiO${}_{3}$ thin films grown on AuNPs showed regions with strongly increased conductivity even in the initial state (Figure 3c). Therefore, these conducting regions can be identified with preformed filaments induced in the thin films by the presence of the AuNPs. Comparing the current scale in the different resistive states depicted in Figure 3c–e, it can be seen that the current level of the initial state was rather in the order of the HRS than in the order of the LRS. Therefore, we suggest that the initial samples contain slightly oxygen-deficient regions, which are further reduced after switching to the LRS. In order to reveal the relationship of these preformed regions to the AuNPs observed at the surface of the SrTiO${}_{3}$ thin films, the conductivity scans in Figure 3c–e were compared with the corresponding topography of the films characterized by SEM and AFM, respectively.

The SEM image in Figure 3a exhibits rectangular structures (blue) with the AuNPs (red) visible as the bright spot. Both the rectangular structure and the Au nanoparticle are clearly observable in the LC-AFM topography image in Figure 3b. Simultaneously with the topography, current scans were recorded for the initial state (Figure 3c), the LRS (Figure 3d) and the HRS (Figure 3e). The area of the AuNP (marked in red) exhibited an increased current flow in the initial state compared to the rectangular structure and the undisturbed SrTiO${}_{3}$ thin film. After scanning with positive bias over the entire scan size, a further increased current flow (note the different current scales) was present in the area of the AuNP (marked in red), whereas the surrounding area remained highly insulating. After scanning with negative bias, there was no measurable current through the film in the AuNP region, which indicates that the AuNP was disconnected from the bottom electrode and not distinguishable from the surrounding SrTiO${}_{3}$ region. The case when the current through the AuNP was changed during switching will be referred as Type I switching in the following.

**Figure 4:**
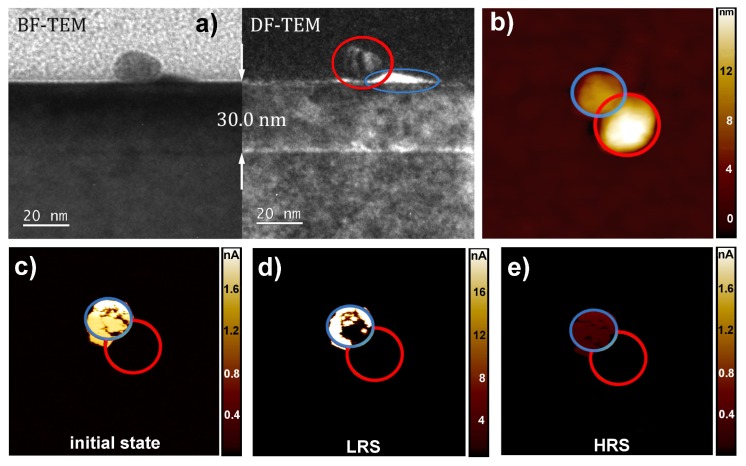
Type II resistive switching at the distorted SrTiO${}_{3}$ island next to the AuNP. (**a**) Bright field (BF) and dark field (DF) TEM images with distorted SrTiO${}_{3}$ island (blue) and AuNP (red) on top of the 30-nm stoichiometric SrTiO${}_{3}$ thin film (identical to Figure 2a). (**b**) Topography image of a 30-nm stoichiometric SrTiO${}_{3}$ thin film deposited at 1.02 J/cm${}^{2}$, containing both the distorted SrTiO${}_{3}$ island and the AuNP. (**c**–**e**) Current images measured with a read out voltage of +0.5 V in the same area as (b). For the initial (c), the LRS after (a) scan with +2 V (d) and the HRS after a scan with −2 V (e). All LC-AFM images were taken with a scan size of 400 nm × 400 nm.

Figure 4 shows a different scenario, which we will refer to as Type II switching in the following. In this scenario, the current flow was not changed across the AuNP itself, as in the case of Type I switching, but next to it. The AFM topography depicted in Figure 4b consists of two elevated structures. The related TEM images depicted in Figure 4a indicate a distorted SrTiO${}_{3}$ island (blue) with a height of around 9 nm next to the AuNP (red). Therefore, the two elevated structures in the AFM topography scan Figure 4b can be attributed to the SrTiO${}_{3}$ island (blue) and the AuNPs (red), respectively. Simultaneously with the topography (Figure 4b), current scans were measured for the initial state (Figure 4c), the LRS (Figure 4d) and the HRS (Figure 4e).

The area of the distorted SrTiO${}_{3}$ island next to the AuNP exhibited an increased conductivity in the initial state, whereas no significant current flow across the AuNP itself and the undisturbed SrTiO${}_{3}$ thin film could be measured. After a scan with positve bias over the entire scan size, most of the distorted SrTiO${}_{3}$ area exhibited a further increased conductivity (note the different current scales), whereas no current flowed across the surrounding SrTiO${}_{3}$ and the AuNP. After a switching scan with negative bias, a slightly lower current than in the initial state was measured across the distorted SrTiO${}_{3}$ area. Although the distorted SrTiO${}_{3}$ island appeared to be distinguishable from the surrounding, the difference was very small and may not be detectable with a slightly less conductive tip.

## 3. Discussion

Although resistive switching occurs at different positions in the vicinity of the AuNPs for Type I and Type II, the polarity and the required voltages are identical. This suggests that both types have the same physical origin. Based on the fact that a positive applied voltage results in the LRS and a negative bias in the HRS, the switching behavior can be identified as the so-called “eightwise switching” [28], which was recently revealed as an oxygen evolution and incorporation process [29,30]. Since we could identify the switching region for the Type II scenario as the distorted SrTiO${}_{3}$ island next to the AuNP, we suggest that in the case of Type I switching, the distorted SrTiO${}_{3}$ underneath the AuNP changes its resistance and thereby electrically connects and disconnects the AuNP with the bottom electrode.

Due to the increased amount of oxygen vacancies in the distorted SrTiO${}_{3}$ regions nearby the AuNPs, the local oxygen evolution may be promoted compared to the surrounding SrTiO${}_{3}$ regions, similar to the observation of an increased oxygen exchange rate at grain boundaries [34]. The existence of a three-phase-boundary among the distorted SrTiO${}_{3}$, the AuNP and the atmosphere may further promote the local oxygen evolution [35]. The locally-enhanced oxygen evolution leads to an increase of the charge carrier concentration, which results in the observed LRS in the current maps for Type I and Type II during positive biasing. During switching with negative bias, the oxygen is reincorporated into the SrTiO${}_{3}$, which results in the observed HRS. This HRS exhibits a lower conductivity than the initial state, which indicates that there may be more oxygen reincorporated than was removed during switching to the LRS.

For both types of switching, a distorted SrTiO${}_{3}$ area is present, which is either beneath (Type I) or next to (Type II) the AuNP. It must be noted that the resistance of the AuNP itself does not change for Type I switching. Instead, the distorted SrTiO${}_{3}$ switches, which electrically connects and disconnects the AuNP from the bottom electrode during the tip-induced redox-reaction.

Enhanced switching has been previously observed in oxide thin films containing defective island boundaries [36] or grain boundaries [14]. However, these defects extended over the whole film thickness and might enhance both the oxygen exchange reaction, as well as oxygen bulk diffusion. It is therefore significant to note that we identified pure surface modifications, which mainly influence the surface exchange rate, as sufficient to enhance the resistive switching properties. Therefore, local surface modification might be a possible novel path to pinpoint the filament position in memristive devices. Our method to use AuNPs as a template for thin film growth represents one possible route along this line of surface-induced filament engineering.

## 4. Materials and Methods

### 4.1. Formation of AuNPs

The chemical approach utilized in this work consists of the electrostatic immobilization of citrate-stabilized AuNPs on (3 aminopropyl) trimethoxysilane (APTMS)-functionalized Nb:STO surfaces [37]. After annealing the 0.5 wt%-Nb-doped SrTiO${}_{3}$ (Nb:STO) single crystal substrates for 4 h at 950 ${}^{\circ }$C in a furnace, the surface was cleaned and activated by O${}_{2}$-plasma and subsequently rinsed in H${}_{2}$O, resulting in OH groups on the surface. These OH groups enable the chemisorption of APTMS molecules on the Nb:STO surface, leading to the formation of a self-assembled monolayer with positively-charged terminal ammonium (NH${}_{4}^{+}$) groups and thus an APTMS-functionalized Nb:STO surface. Citrate-stabilized AuNPs with a diameter of approximately 15 nm and a negative zeta-potential were obtained by the Turkevich method [38]. These AuNPs were deposited from solution onto the APTMS-functionalized Nb:STO surface. The opposing charges of the APTMS-functionalized surface and the citrate-stabilized AuNPs lead to the electrostatic immobilization of the AuNPs [39]. The citrate components were removed from the Nb:STO substrates after AuNP immobilization in two steps. Firstly, the substrates were cleaned by O${}_{2}$-plasma with 100 W and 200 mL/min, O${}_{2}$ for 5 min. Secondly, the substrates were annealed for 20 min in the deposition chamber at 800 ${}^{\circ }$C in 0.1 mbar O${}_{2}$ [40]. The temperature during this cleaning process is a critical parameter, since the melting point of AuNPs is lower for smaller nanoparticle diameters and coalescence must be avoided [41,42]. The AuNPs used in this work, which have a diameter of approximately 15 nm, withstood this cleaning process.

### 4.2. SrTiO${}_{3}$ Thin Film Growth

SrTiO${}_{3}$ thin films were prepared by pulsed laser deposition (cluster system of Surface Inc.) with 0.1 mbar O${}_{2}$, a substrate temperature of 800 ${}^{\circ }$C and a laser fluence of 1.02 J/cm${}^{2}$, which resulted in stoichiometric SrTiO${}_{3}$ thin films [25], with high crystalline quality.

### 4.3. Atomic Force Microscopy

In this work, an Omicron VT-SPM system by Omicron Nano Technology GmbH was used for the simultaneous measurement of topography and current. The measurements were performed in contact mode, under ultra high vacuum (UHV) conditions (10${}^{-10}$ mbar) at room temperature. Two types of cantilevers were used, namely single-crystalline diamond (“Dia”, NaDiaProbes from nanoScience instruments) and doped diamond (“UNCD”, DopedDiamond from AppNano). All LC-AFM measurements were performed in situ after pulsed laser deposition to keep contaminations at a minimum.
